# Painless Tooth Extraction

**Published:** 1886-02

**Authors:** 


**Painless Tooth Extraction.**—Dr. Hepburn, in the *Independent Practioner*, says that teeth can be extracted without pain in the following manner: The tincture of purified extract of canabis indica is diluted with from three to five parts of water. This is applied to the gums by rubbing with the finger dampened with the solution. The forceps are also dipped into the solution before applying them to the teeth.—*N. E. Medical Monthly.*

**Crude** honey has been found to keep far better than the clarified kind but the addition of about one per cent. of oxalic acid will do no harm to the honey, but will improve it.

